# Tapering of biological antirheumatic drugs in rheumatoid arthritis patients is achievable and cost-effective in daily clinical practice: data from the Brussels UCLouvain RA Cohort

**DOI:** 10.1186/s13075-020-02165-4

**Published:** 2020-04-28

**Authors:** Stéphanie Dierckx, Tatiana Sokolova, Bernard R. Lauwerys, Aleksandra Avramovska, Laurent Meric de Bellefon, Adrien Nzeusseu Toukap, Maria Stoenoiu, Frédéric A. Houssiau, Patrick Durez

**Affiliations:** Rheumatology, Cliniques universitaires Saint-Luc - Université catholique de Louvain - Institut de Recherche Expérimentale et Clinique (IREC), Brussels, Belgium

**Keywords:** Rheumatoid arthritis, bioDMARDs, Remission, Dose tapering

## Abstract

**Background/purpose:**

Studies have demonstrated that rheumatoid arthritis (RA) patients who achieve low disease activity or remission are able to taper biological disease-modifying antirheumatic drugs (bDMARDs). The aim of this study was to evaluate the proportion of patients in whom bDMARDs can be tapered in daily practice and to analyse the characteristics of these patients. Other objectives were to analyse which bDMARDs are more suitable for dose reduction and the cost savings.

**Results:**

Data from 332 eligible RA patients from our Brussels UCLouvain cohort were retrospectively analysed; 140 patients (42.1%) received a tapered regimen, and 192 received stable doses of bDMARDs. The age at diagnosis (43.1 vs 38.7 years, *p* = 0.04), health assessment questionnaire (HAQ) score (1.3 vs 1.5, *p* = 0.048), RF positivity rate (83.3 vs 72.9%, *p* = 0.04) and disease duration at the time of bDMARD introduction (9.7 vs 12.1 years, *p* = 0.034) were significantly different between the reduced-dose and stable-dose groups. Interestingly, relatively more patients receiving a tapered dose were treated with a combination of bDMARDs and methotrexate (MTX) (86.7% vs 73.8%, *p* = 0.005). In our cohort, anti-TNF agents were the most commonly prescribed medications (68%). Only 15 patients experienced a flare during follow-up. Adalimumab, etanercept and rituximab were the most common bDMARDs in the reduced-dose group and were associated with the most important reductions in annual cost.

**Conclusion:**

In daily practice, tapering bDMARDs in RA patients who have achieved low disease activity or remission is an achievable goal in a large proportion of patients, thereby reducing potential side effects and annual drug-associated costs. The combination of bDMARDs with MTX could improve the success of dose reduction attempts.

**Trial registration:**

This retrospective non-interventional study was retrospectively registered with local ethics approval.

## Introduction

Rheumatoid arthritis (RA) can lead to major deformities and loss of function, especially in the absence of a clinical response. Biological disease-modifying antirheumatic drugs (bDMARDs) have considerably improved the prognosis of RA. In clinical practice, these agents have led to remission or low disease activity (LDA) in many RA patients.

Care strategies have been developed, including the “treat to target” approach recommended by the European League Against Rheumatism (EULAR), which suggests initiating treatment quickly once the diagnosis is established and adapting it until remission or LDA is reached [1, 2].

Subsequently, the question has arisen as to whether long-term treatment with the full dose of bDMARDs is necessary in patients who achieve the objective of remission or LDA.

The strategy to reduce the doses of bDMARDs has potentially beneficial effects in several areas, such as the risk of side effects, especially infections; the comfort of the patient; and the economic impact of the drug [2, 3].

Several studies have reported that tapering bDMARDs is an achievable goal in RA patients [4]. At this time, there is a lack of data to support this strategy in daily clinical care.

The objectives of this study were to assess whether bDMARDs dose reduction is feasible in daily practice and to analyse the associated disease and patient characteristics. In a second step, we calculated the proportion of patients who were able to benefit from dose reduction stratified by the specific bDMARD used and assessed the economic impact of dose reduction for each bDMARD.

## Methods

### Study design

This was a retrospective study of 332 RA patients fulfilling the 1987 and/or 2010 RA classification criteria; the data were analysed in December 2017. To be included, patients must have received the same bDMARD therapy for at least 1 year and received follow-up care in our Louvain Clinic from 2000 to 2018. A reduction in the dose of the last and current bDMARD was proposed before 2017 by a senior physician in our UCLouvain Rheumatology Department for patients who achieved the absence of synovitis on clinical examination with sustained LDA or remission for 6 months.

RA patients were divided into 2 groups: a group of patients who still received bDMARDs at the standard dose and a second group who received bDMARDs at reduced doses.

We evaluated the following patient and disease characteristics: the age at RA diagnosis, age at the introduction of the current biological, age at the time of the study, sex, smoking status, presence of rheumatoid factor (RF) or citrullinated antipeptide antibodies (ACPA), presence or absence of radiological erosion, duration of the disease at the introduction of the first conventional synthetic DMARD (csDMARD) and bDMARD and the number of bDMARDs received. Several clinical variables evaluated at the introduction of the current bDMARD treatment were also collected: the patient global assessment (PGA) score, Health Assessment Questionnaire (HAQ) score, CRP level, number of tender and swollen joints, Disease Activity Score-28 for Rheumatoid Arthritis with CRP (DAS28-CRP) and concomitant intake of MTX and/or glucocorticoids. Finally, we noted the latest DAS28-CRP encoded at the inclusion in December 2017.

### Endpoints

We calculated the proportion of patients who were treated with a reduced bDMARD dose and compared it with the proportion who remained on a stable dose. We then compared the characteristics of these patients (age at diagnosis, age at the introduction of the current bDMARD, sex, smoking status, presence or absence of erosion, RF and ACPA) and activity index scores at the introduction of the current bDMARD (visual analogue scale (VAS) score, HAQ score, number of painful or swollen joints and DAS28-CRP). For each of the groups, we also calculated the percentage of patients receiving methotrexate and/or glucocorticoids when the current bDMARD was introduced.

We determined the suitability of dose reduction for each bDMARD. We also analysed the percentage dose reduction (< 50%, 50% or > 50%) and the number of retrospective relapses observed in the “reduced-dose” group, defined by a flare (presence of synovitis and DAS28-CRP above 3.2) and the reinitiation of the full dose. For each bDMARD, we calculated the annual cost per patient when the dose was reduced and assessed the economic impact of the dose reduction.

### Statistical analysis

The descriptive data are expressed as the average ± SD (95% CI) or the percentage. We used Levene’s test to analyse the equality of variance. The Mann-Whitney test (if the variance was not equal) or Student’s *t* test (if the variance was equal) were used to compare the following variables: the age at diagnosis, age at the introduction of the current biologic therapy, duration of disease at the introduction of the first csDMARD or bDMARD, number of swollen joints, number of tender joints, VAS score, HAQ score and DAS28-CRP. Chi-square test was used to compare the following variables: sex, smoking status, the presence of ACPA, the presence of RF, erosion, glucocorticoid intake and methotrexate intake. A *p* value < 0.05 was considered statistically significant. SPSS Statistics 25 software was used.

## Results

### Patient population

**Fig. 1:**
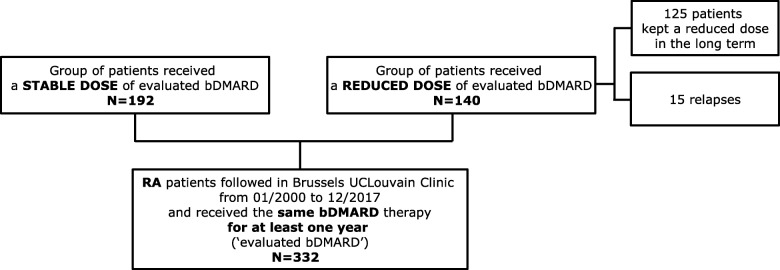
Retrospective design trial profile

A total of 332 patients were retrospectively analysed in the study; 192 (57.9%) were treated with a stable dose of bDMARDs, and 140 (42.1%) were treated with a reduced dose (Fig. 1). In 125 patients, a reduced dose of the current bDMARD was maintained during follow-up (mean duration of 14.6 ± 6.6 years), and 15 patients experienced a relapse that justified closer interval between doses or a dose increase.

### Characteristics of the study population and baseline features before the introduction of the current biological treatment

Patients in the reduced-dose group were significantly older than those in the stable-dose group (60.7 vs 55.7 years, *p* = 0.02) and were also significantly older at diagnosis (43.1 vs 38.7 years, *p* = 0.04). There was no statistically significant difference between the groups regarding ACPA status, but there were more patients with RF in the reduced-dose group than in the stable-dose group (83.3 vs 72.9%, *p* = 0.04). The proportions of patients with erosion were similar, and there was no significant difference according to sex. There was no statistically significant difference in disease duration since the introduction of the first csDMARD or bDMARD treatment, but the duration of disease at the introduction of the current bDMARD treatment was significantly shorter in the reduced-dose group than in the stable-dose group (9.7 vs 12.1 years, *p* = 0.034) (Table 1).

**Table 1:** Characteristics of the study population and their disease before the evaluated BIO DMARDs administration

	Total group (*n* = 332)	Standard care group (*n* = 192)	Dose reduction group (*n* = 140)	*p* value
Patient age in 2017, years (mean ± SD)	57.83 ± 15.12	55.70 ± 15.78	60.74 ± 13.69	0.02
Patient age at the RA diagnostic, years (mean ± SD)	40.56 ± 13.89	38.72 ± 13.99	43.08 ± 13.39	0.04
Patient age at the introduction of the evaluated biologic treatment, years (mean ± SD)	51.16 ± 14.33	49.99 ± 14.82	52.77 ± 13.50	
Disease duration at the introduction of the first sDMARD, years (mean ± SD)	2.10 ± 5.12	2.05 ± 5.21	2.17 ± 5.02	
Disease duration at the introduction of the first bDMARD, years (mean ± SD)	8.33 ± 9.13	9.06 ± 9.62	7.34 ± 8.35	
Disease duration at the introduction of the evaluated bDMARD, years (mean ± SD)	11.09 ± 9.99	12.08 ± 10.60	9.73 ± 8.95	0.034
Women, *n* (%)	259 (78%)	143 (74.5%)	116 (82.9%)	
Anticyclic citrullinated peptide antibody positive, *n* (%)	221 (73.9%)	123 (70.3%)	98 (79%)	
Rheumatoid factor positive, *n* (%)	252 (77.3%)	137 (72.8%)	115 (83.3%)	0.04
Presence of erosion, *n* (%)	290 (87.3%)	166 (86.5%)	124 (88.6%)	
Smoking status, *n* (%)	52 (17%)	28 (16,4%)	24 (17.6%)	
Tender joint count (0–68 scale) at the introduction of the evaluated bDMARD (mean ± SD)	11.02 ± 8.8	11.14 ± 8.52	10.85 ± 9.33	
Swollen joint count (0–68 scale) at the introduction of the evaluated bDMARD (mean ± SD)	8.56 ± 5.77	8.41 ± 6.06	8.76 ± 5.36	
Health assessment questionnaire (0–3 scale) at the introduction of the evaluated bDMARD (mean ± SD)	1.45 ± 0.71	1.52 ± 0.70	1.34 ± 0.71	0.048
Patient global assessment (0–100 mm) at the introduction of the evaluated bDMARD (mean ± SD)	64.29 ± 23.70	67.11 ± 22.33	60.09 ± 25.14	0.024
C-reactive protein (mg/dl) at the introduction of the evaluated bDMARD (mean ± SD)	2.61 ± 7.19	2.71 ± 9.08	2.49 ± 3.21	
Disease activity score in 28 joints at the introduction of the evaluated bDMARD (mean ± SD)	4.82 ± 1.02	4.83 ± 0.98	4.80 ± 1.09	
Glucocorticoids intake at the introduction of the evaluated bDMARD, *n* (%)	173 (53.2%)	99 (52.1%)	74 (54.8%)	
Methotrexate intake at the introduction of the evaluated bDMARD, *n* (%)	258 (77.7%)	141 (73.8%)	117 (86.7%)	0.005

In addition, we noted that there were proportionately more patients treated concurrently with MTX in the reduced-dose group than in the stable-dose group, and the difference was highly significant (86.7% vs 73.8%, *p* = 0.005). There was no difference in glucocorticoid intake between the two groups. The HAQ and PGA scores at the introduction of the current bDMARD treatment were significantly lower in the reduced-dose group than in the stable-dose group (1.3 vs 1.5, *p* = 0.048 and 60.1 vs 67.1, *p* = 0.024, respectively). There was no statistically significant difference in the DAS28-CRP at baseline (Table 1).

### Comparison of the DAS28-CRP between the 2 groups

As expected, the DAS28-CRP recorded at the last visit was higher in the group that did not benefit from a dose reduction, indicating more active disease, than in the group that benefited from a dose reduction (2.64 vs 2.26, *p* = 0.001).

### Analysis according to the different types of received biological treatments, dose reduction and relapses

Patients in the stable-dose group received a greater number of different bDMARDs than those in the reduced-dose group, as shown in Table 2. This likely reflects a better primary response to bDMARD in the reduced-dose group than in the stable-dose group (Table 2).

**Table 2:** Previous number of bDMARDs administered

Number of bDMARDs received	Total group % (*n*)	Standard care group % (*n*)	Dose reduction group % (*n*)
**1**	**53.3** (177)	**49.5** (96)	**57.6** (81)
**2**	**29.5** (98)	**32.1** (61)	**26.6** (37)
**3**	**8.4** (28)	**8.3** (16)	**8.5** (12)
**4**	**5.7** (19)	**6.3** (12)	**5.0** (7)
**5**	**2.4** (8)	**2.6** (5)	**2.2** (3)
**6**	**0.6** (2)	**1.1** (2)	**0** (0)
**Total**	**100** (332)	**100** (192)	**100** (140)

Anti-TNFs were the most frequently prescribed bDMARDs, with a total of 68% of the patients receiving them (29% of the patients received infliximab (IFX), 18% received etanercept (ETN), 13% received adalimumab (ADA), 7% received golimumab (GOL), and 1% received certolizumab (CZP)). The non-TNFi bDMARDs prescribed were tocilizumab (TCZ) (15%), rituximab (RTX) (10%) and abatacept (ABA) (7%).

**Fig. 2:**
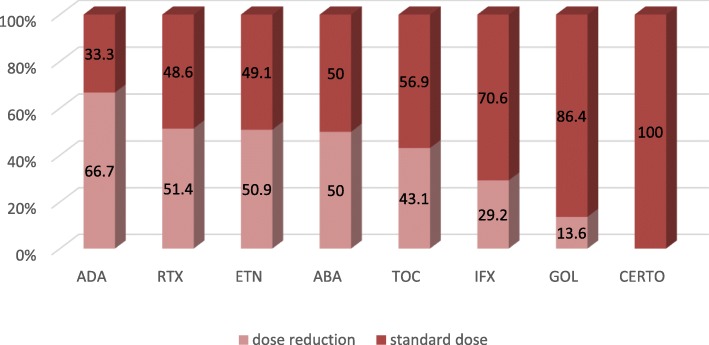
Proportion of patients with decreased dose for each bDMARD. ABA abatacept, ADA adalimumab, CZP certolizumab, ETN etanercept, GOL golimumab, IFX infliximab, RTX rituximab, TOC tocilizumab

ADA, ETN and RTX were the most common bDMARD in the reduced-dose group (Fig. 2). Indeed, 66.7% of the patients who received ADA benefited from a reduction of the dose; furthermore, 51.4% of the patients who received RTX, 50.8% of the patients taking ETN, 50% of the patients taking ABA, 43.1% of the patients taking TCZ, 29.2% of the patients taking IFX and 13.6% of the patients taking GOL benefited from a dose reduction. No patient benefited from a reduced dose in the CZP group, but only 5 patients were included, which limits the interpretation.

Of the 140 patients in the reduced-dose group, 11 patients were able to reduce the biological treatment dose by more than 50%, 39 were able to reduce it by 50% and 75 were able to reduce it by less than 50%. In addition, 15 patients out of 140 (10.7%) experienced a relapse after a mean period of 1.9 ± 1.5 years (min 0.5–max 2.8 years) that necessitated a closer interval between doses or an increase in the bDMARD dose.

### Economic impact of reducing the dose of biological treatments

The annual cost per patient was significantly reduced for those taking RTX and anti-TNF agents, mainly ADA and ETN. Indeed, for patients taking these drugs, the annual cost was reduced by nearly 50%: for RTX, the annual cost per patient was 8784 euros in the stable-dose group and 4675 euros in the reduced-dose group; for ETN, it was 9328 euros in the stable-dose group and 5580 euros in the reduced-dose group; and for ADA, the cost was 12,525 euros in the stable-dose group and 7175 euros in the reduced-dose group. We do not have data for GOL and CZP because of the small number of patients in those groups. IFX had the smallest reduction in the annual cost, i.e., from 7290 euros in the standard dose group to 6146 euros in the reduced-dose group, which corresponds to a reduction of 15% (Table 3).

**Table 3:** Annual cost in euros per patient and per bDMARD

bDMARD	Standard care group *n* (%)	Annual cost per patient in euros (€) for the standard care group	Dose reduction group *n* (%)	Annual cost per patient in euros (€) for the dose reduction group
ABA	11 (5.7)	**12,979**	11 (7.9)	**8643.5**
ADA	14 (7.3)	**12,525**	28 (20.0)	**7175.03**
CZP	5 (2.6)	**11,740.2**	0 (0)	**Not available**
ETN	29 (15.1)	**9328.6**	30 (21.4)	**5580.04**
GOL	19 (9.9)	**12,703.08**	3 (2.1)	**Not available**
IFX	68 (35.41)	**7290**	28 (20)	**6146.5**
RTX	17 (8.85)	**8784**	18 (12.85)	**4675.03**
TOC	29 (15.1)	**12,773.7**	22 (15.71)	**9487.7**
	192		140	

## Discussion

Our study is one of the first to demonstrate that dose reduction of bDMARDs is feasible in daily clinical practice and in standard of care. Various studies have shown that many RA patients can taper bDMARDs and still maintain remission or LDA [5, 6]. One of the main studies that investigated this topic was the PRESERVE study. Smolen reported that the reduction of ETN from 50 to 25 mg was not followed by any loss of efficacy [7]. Similar results were observed in the DOSERA study [8]. In the STRASS trial, which employed a treatment to target strategy with anti-TNF agents, 68.5% of the patients maintained remission or LDA, with sustained efficacy at 3 years observed in 41% [9, 10]. In the DRESS trial, the proportions of patients with relapse and radiological progression did not differ between groups with a reduced or stable dose of ETN or ADA [11, 12]. The withdrawal of bDMARDs has been proposed in several trials and has been mainly evaluated in early RA studies, such as the OPTIMA trial [13–15]. This question was not analysed in our cohort since one of the criteria to be included was to be treated with a bDMARD. Most studies reported data on TNFi, few data are available for non TNFi bDMARDs, except a small retrospective cohort on TCZ. More recently, equivalent therapeutic maintenance has been observed between 4 and 2 mg per day baricitinib, which is a JAK inhibitor [16]. Clinicians are interested in determining the profile of patients who are likely to successfully maintain remission or LDA when taking a reduced dose of bDMARDs. Our study showed that different baseline RA characteristics were correlated with the success of the bDMARD dose decrease, such as the age at diagnosis, presence of RF, disease duration at the introduction of the first bDMARD, HAQ score, PGA score and combination with MTX. Relatively more older patients received a reduced dose, which could be explained by a potential decrease in the severity of RA with age and also by the clinician’s willingness to reduce the dose to reduce the risk of potential side effects, especially infections, in the relatively older population. Another parameter that was significantly correlated with the success of the dose reduction was a shorter disease duration at the time of the introduction of the current bDMARD, which may be explained by a reduced response to bDMARD therapy observed with disease duration, progressive structural damage and a greater number of comorbidities [17, 18]. One interesting finding of our study was that in daily practice, the concomitant use of MTX could improve the success of bDMARD dose reduction attempts. The combination of MTX with bDMARDs is supported by a number of bDMARD studies, including data from the national registry and the international recommendations for the treatment of RA [1–3, 19]. We could not exclude the possibility that physicians were more prone to decrease bDMARD doses in patients treated with MTX. In daily practice, clinicians, patients and payers are interested in determining in which bDMARD dose reduction is more likely to be successful. Our data indicate that ETA and ADA are the drugs best suited for dose reduction after LDA or remission status is achieved. This could be explained by the large number of patients treated with these drugs as well as a longer follow-up. Other bDMARDs, such as IFX, ABA or TCZ, could also potentially be reduced but to a lesser extent. The success of the reduction in dose in some bDMARDs is potentially explained by the half-life of the treatment and the recorded dose. Indeed, for IFX, the reimbursed doses are only 3 mg per kilogram every 8 weeks in RA patients, so it is difficult to reduce the already low dose, exposing the patient to the risk of developing anti-drug antibodies. Interestingly, we report that the doses of RTX can be widely spaced over time, which could be explained by the sustained effect of RTX over time in good responders. In our cohort, 11, 39 and 75 patients were able to reduce the biological treatment dose by more than 50%, 50% and less than 50%, respectively. Our data are comparable with that obtained in a cohort study of routine care RA patients in Denmark that reported a 28% chance of successfully reducing the bDMARDS dose by half [14]. A previous study has reported a correlation between ACPA positivity and disease relapse [13]. In our study, no analysis was possible since only 15 patients experienced relapse. Substantial cost savings can be achieved if rheumatologists select biosimilars [20]. We analysed the annual cost of bDMARDs in our cohort and demonstrated that the annual cost savings (percentage reduction) was estimated to be nearly 40% without taking into account other factors affecting the cost, such as the number of infusions, nursing care for injections and the potential reduction in infectious complications. Cost savings were published in the DRESS study and in an IFX study [21, 22]. A recent study performed in Denmark reported an accumulated cost reduction of 4,178,000 € in 997 bDMARD cases (23).

However, our study has important limitations, such as the retrospective nature of the analysis, the variability in the bDMARDs used and the dose reductions and potential confounders such as the disease severity or patient perspectives. We therefore suggest further prospective analyses using national registers.

## Conclusions

The dose reduction of bDMARDs in RA patients is achievable in current practice when disease control is observed. The combination of bDMARDs with MTX could improve the success of bDMARD dose reduction attempts. ADA, ETN and RTX were the most frequently reduced bDMARDs in our cohort. A large economic benefit related to the dose reduction of bDMARDs as represented by the annual cost per patient was confirmed. Further large prospective trials in daily clinical practice are needed to confirm the benefits of this approach for patients, physicians and payers.

## Data Availability

All data were collected in medical record files of Cliniques universitaires Saint-Luc, Brussels, Belgium.
